# Institutional injustice: Implications for system transformation emerging from the mental health recovery narratives of people experiencing marginalisation

**DOI:** 10.1371/journal.pone.0250367

**Published:** 2021-04-16

**Authors:** Ada Hui, Stefan Rennick-Egglestone, Donna Franklin, Rianna Walcott, Joy Llewellyn-Beardsley, Fiona Ng, James Roe, Caroline Yeo, Emilia Deakin, Sarah Brydges, Patricia Penas Moran, Rose McGranahan, Kristian Pollock, Graham Thornicroft, Mike Slade

**Affiliations:** 1 School of Health Sciences, Institute of Mental Health, University of Nottingham, Nottingham, United Kingdom; 2 NEON Lived Experience Advisory Panel, Nottingham, United Kingdom; 3 Department of Digital Humanities, King’s College London, London, United Kingdom; 4 National Institute for Health Research, ARC East Midlands, University of Nottingham, Nottingham, United Kingdom; 5 School of Pharmacy, University of Nottingham, Nottingham, United Kingdom; 6 Department of Personality, Assessment and Psychological Treatment, University of Deusto, Bilbo, Spain; 7 Unit of Social and Community Psychiatry, Queen Mary University of London, London, United Kingdom; 8 School of Health Sciences, University of Nottingham, Nottingham, United Kingdom; 9 Centre for Global Mental Health and Centre for Implementation Science, Health Service and Population Research Department, Institute of Psychiatry, Psychology and Neuroscience, King’s College London, London, United Kingdom; University of Edinburgh, UNITED KINGDOM

## Abstract

**Background:**

Institutional injustice refers to structures that create disparities in resources, opportunities and representation. Marginalised people experience institutional injustice, inequalities and discrimination through intersecting personal characteristics and social circumstances. This study aimed to investigate sources of institutional injustice and their effects on marginalised people with experience of mental health problems.

**Methods:**

Semi-structured interviews were conducted with 77 individuals from marginalised groups with experience of mental health problems, including psychosis, Black, Asian and minority ethnic (BAME) populations, complex needs and lived experience as a work requirement. These were analysed inductively enabling sensitising concepts to emerge.

**Findings:**

Three processes of institutional injustice were identified: not being believed because of social status and personal backgrounds; not being heard where narratives did not align with dominant discourses, and not being acknowledged where aspects of identity were disregarded. Harmful outcomes included disengagement from formal institutions through fear and mistrust, tensions and reduced affiliation with informal institutions when trying to consolidate new ways of being, and damaging impacts on mental health and wellbeing through multiple oppression.

**Conclusions:**

Institutional injustice perpetuates health inequalities and marginalised status. Master status, arising from dominant discourses and heuristic bias, overshadow the narratives and experiences of marginalised people. Cultural competency has the potential to improve heuristic availability through social understandings of narrative and experience, whilst coproduction and narrative development through approaches such as communities of practice might offer meaningful avenues for authentic expression.

## Introduction

The tradition in mental health research has been to focus on individuals: their engagement with professionals, adherence to treatment and the effectiveness of interventions to alleviate their symptoms [1]. The influences of institutions on individuals has been a primary focus for sociology, but has not been the main lens within psychiatry, leading to an ‘interdisciplinary void’ [2]. Institutional structures, policies and practices typically serve the needs of majority populations to the detriment of non-majority groups [3], so addressing this interdisciplinary void is important [4].

Institutional structures create injustice where there are disparities in resources, opportunities and representation between majority and non-majority perspectives [5] for example, inequalities in access to treatment, experiences of health services and treatment outcomes [6, 7] Majority and minority populations refer to relative group size, membership and social status. Non-majority populations, here called *marginalised communities*, are particularly affected by multiple disadvantage [8].

Marginalised communities are disadvantaged, under-represented and under-served by institutions, and are more likely to experience institutional injustice [9]. Individuals from marginalised communities experience inequalities and discrimination relating to intersecting personal characteristics, such as gender, ethnicity and sexual orientation, along with disparities of social capital [10], such as poverty, education, employment and accommodation [5]. Marginalised people participating in this study include those from Black, Asian and minority ethnic (BAME) communities, lesbian, gay, bisexual, transgender or queer (LGBTQ+) communities, people experiencing mental health problems choosing not to use mental health support and those with complex needs including substance misuse and/or homelessness [11]. Additionally, peer support workers continue to report stigma and discrimination due to intersecting characteristics between their lived experiences and work roles [12, 13].

For individuals whose personal and social characteristics intersect in ways that result in marginalisation, experiencing institutional injustice is more likely, making their journeys of recovery consequently more challenging [14]. Policies based on evidence derived from majority populations reinforce dominant discourses through bias and under-representation of non-majority populations, having the effects of further excluding non-majority populations [15]. Daily practices guided by these processes exacerbate health and social inequalities through discriminatory biases [16].

Injustice caused by institutions lead to unintended harms being experienced; a notion we refer to as institutional injustice. *Institutional injustice* is defined as the inadvertent harms caused by institutions to the individuals they seek to serve, even when the professed intentions underpinning the institution are benevolent. Institutional injustice has been considered in the context of violence and coercion, but less often as a lens by which to consider the processes and outcomes relating to peoples’ experiences of mental health [17].

People living with mental health problems may experience injustice across multiple formal and informal institutions. *Formal institutions* are those governed by the state, regulatory bodies, policies and practices, including inpatient hospitals and community based mental health services. *Informal institutions* comprise socially organised groups, such as families, communities, social networks and relationships [18]. Institutions have varied agendas, discourses and priorities, each of which impact on how institutions explain or understand experiences, and how people using the institution feel, behave or respond [19]. The perspectives of marginalised communities are often systematically excluded from shaping policy and practice [20], resulting from a lack of meaningful engagement in research, dialogue, participation, outcomes and policy recommendations [21]. Effects of institutional injustice include reduced engagement with mental health services, reduced treatment adherence and reduced effectiveness of mental health service intervention [22], which may be costly to the individual in terms of their health and wellbeing, quality of life and mortality [23].

The aim of this study was to develop a model describing how institutional injustice creates negative change, to i) identify processes of institutional injustice; ii) investigate their effects on the person and iii) explore implications for institutional change.

## Methods

This paper reports on data collected between March and August 2018 as part of the Narrative Experiences Online (NEON) programme (researchintorecovery.com/neon). Ethical approval was granted for this study by the Nottingham 2 Research Ethics Committee (REC Reference: 17/EM/0401). All participants provided written informed consent to take part in the NEON study.

### Participants

77 participants were included in the study (see Table 1). Inclusion criteria common to all groups were: aged 18 years and over; willing to discuss personal experiences of recovery; fluent in English; able to give informed consent. Participants were from four marginalised groups. Group A comprised people having self-identified experiences of psychosis and no use of secondary mental health services over the previous five years. Group B comprised Black and Minority Ethnic (BAME) mental health service users. Group C comprised people who are not well engaged with by mental health services, including LGBTQ+, multiple complex needs, rural communities. Group D comprised people with experience of working in statutory or voluntary roles for which lived experience is a requirement and who are more likely to experience stigma and discrimination due to intersecting characteristics between their lived experiences and work roles, e.g. peer support workers, trainers or researchers.

**Table 1:** Clinical and sociodemographic characteristics of participants (n = 77).

Characteristic	Total	Group A (Outside the system)	Group B (BAME)	Group C (Under-served)	Group D (Peer)
n (%)	77 (100)	21 (27)	21 (27)	19 (25)	16 (21)
**Gender** n (%)					
Female	42 (55)	14 (67)	11 (53)	8 (42)	9 (56)
Male	30 (39)	6 (29)	9 (43)	9 (47)	6 (38)
Other / prefer not to say	5 (6)	1 (5)	1 (5)	2 (11)	1 (6)
**Age** (years)					
18–25	4 (5)	0 (0)	0 (0)	3 (16)	1 (6)
25–34	16 (21)	3 (14)	6 (29)	4 (21)	3 (19)
35–44	16 (21)	5 (24)	4 (19)	4 (21)	3 (19)
45–54	30 (39)	8 (38)	9 (43)	6 (32)	7 (43)
55+	5 (6)	4 (19)	0 (0)	0 (0)	1 (6)
Prefer not to say	6 (8)	1 (5)	2 (10)	2 (11)	1 (6)
**Ethnicity** n (%)					
White British	44 (57)	12 (57)	0 (0)	18 (95)	14 (88)
Black British	5 (6)	2 (10)	3 (14)	0 (0)	0 (0)
Black African / Caribbean	4 (5)	1 (5)	3 (14)	0 (0)	0 (0)
White Other	5 (6)	2 (10)	1 (5)	0 (0)	2 (13)
White and Black African / Caribbean	4 (5)	0 (0)	4 (19)	0 (0)	0 (0)
Asian / Mixed white Asian	4 (5)	0 (0)	4 (19)	0 (0)	0 (0)
Other	5 (6)	2 (10)	3 (14)	0 (0)	0 (0)
Prefer not to say	6 (8)	2 (10)	3 (14)	1 (5)	0 (0)
**Sexual orientation**					
Heterosexual	49 (64)	15 (71)	14 (67)	6 (32)	14 (88)
LGBTQ+	18 (23)	3 (14)	4 (19)	9 (47)	2 (13)
Prefer not to say	10 (13)	3 (14)	3 (14)	4 (21)	0 (0)
**Primary diagnosis**					
Schizophrenia or other psychosis	11 (14)	5 (24)	4 (19)	2 (11)	0 (0)
Bipolar disorder / Cyclothymia	16 (21)	8 (38)	1 (5)	3 (16)	4 (25)
Mood disorder, e.g. anxiety, depression, dysthymia	15 (19)	1 (5)	4 (19)	4 (21)	6 (38)
Other, e.g. ADHD, personality disorder, substance abuse, autism	7 (9)	0 (0)	2 (10)	3 (16)	2 (13)
Prefer not to say	28 (36)	7 (33)	10 (48)	7 (37)	4 (25)

### Setting

Participants were recruited across England; Groups A and B primarily from London and Groups C and D primarily from the Midlands. Recruitment for all groups also used snowball sampling.

### Procedures

Semi-structured interviews were conducted in a health service or community venue to suit participant preference. The topic guide started with a request for participants to share their story of mental health and recovery, with a beginning, a middle and an end. A narrative approach towards the interview allowed scope for individuals to tell their stories in ways they felt most comfortable [24]. Narrative approaches focus on the lives of individuals as told in their own words and through their personal lens. Narrative approaches place emphases on the importance of experiences and meaning, allowing possibilities for understanding experiences and phenomenon through different perspectives [25]. Researcher prompts were used for elaboration and clarity but minimised to allow participants to construct their narratives in their own words. Follow up questions included; who have you shared your story with and why; what was the impact on the recipient and on you, are their aspects of your story you hold back; and has the way you have told your story changed over time. The interviews lasted between 40–90 minutes. They were digitally recorded, professionally transcribed, anonymised and checked for accuracy by the researchers.

### Analysis

Inductive analysis was undertaken using NVivo version 12 Pro. An inductive approach allowed concepts to emerge from participant narratives, rather than from existing frameworks, enabling formulation of concepts and development of ideas [26]. An emphasis on the emergence of concepts being from the participant narratives was particularly important given that people from marginalised communities are not often listened to. Inductive analysis emphasises the importance of emergence of ideas, context and positioning of participants, sensitising the researchers to ‘important features of social interaction…in specific settings’ [27] which are crucial in the study of marginalised experiences. Participant narratives were therefore central to the development and presentation of findings.

Participants reported institutional injustice arising from various sources, in a range of forms and with different effects. These concepts were developed and refined iteratively and inductively by four independent coders (AH, ED, PM and SB). Inductive approaches enabled the coders to identify emerging concepts, whilst iterative approached enabled careful reading and re-reading for further development, refinement and definitions of these concepts. Four transcripts were initially double coded to ensure consistency, followed by continued regular discussions, enabling comprehensiveness and confirmability [28]. Analysis were discussed with wider members of the research team, which included people bringing research, activism and clinical perspectives for quality and rigor. The findings are presented as concepts intended to sensitise the reader to salient phenomena observable in the data, selected for their value in informing institutional policy, practice and transformation [29].

## Results

**Fig 1:**
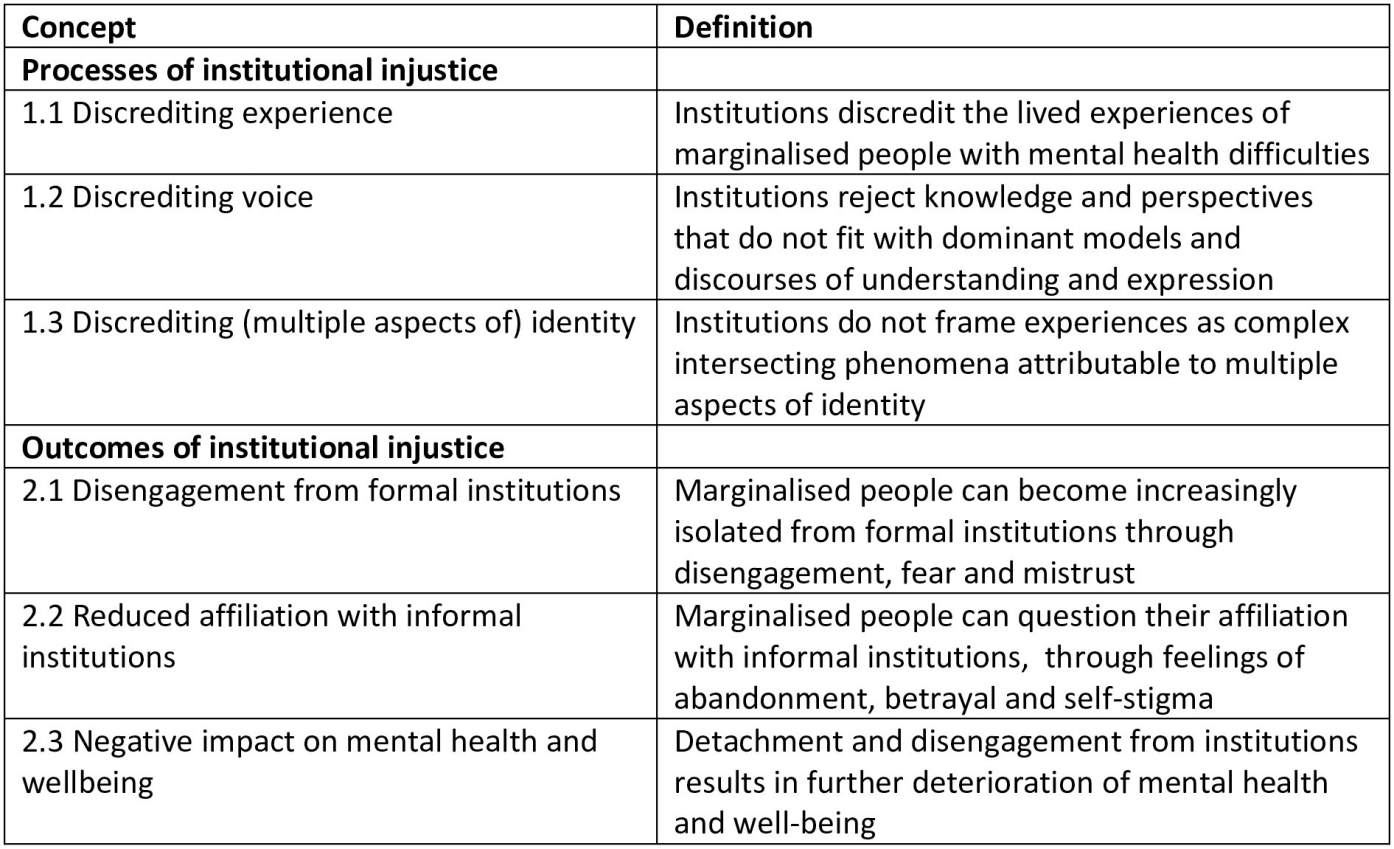
Sensitising concepts on institutional injustice.

A total of 77 qualitative interviews were completed, with participant demographics shown in Table 1. Sensitising concepts identifying processes of institutional injustice and harmful outcomes are shown in Fig 1.

### Processes of institutional injustice

Participants reported processes of discreditation relating to experience, voice and identity.

#### Discrediting experience

Participants reported not having opportunities to voice their experiences and being discredited because of their diagnoses or social status. For some participants, this meant being judged and undermined, or their experiences attributed to blame or disbelief. For example, a participant recalled that because she was a child and because her mother was a prostitute, her experiences were disavowed.

*“So you’ve got an eleven year old sitting in front of you*, *telling you that they’ve been abused*, *and yet you’re sitting there going ‘It didn’t really happen did it*, *you’re just story telling*. *Your mother’s a prostitute*, *your mother’s been in here a few times for arrests so…’ You know*, *yeah*, *that’s the treatment for an eleven year old”*

*(B009*: *Female*, *45–54*, *White and Black African/Caribbean)*

Participants reported being discredited because of their experiences of mental health difficulties and having fundamental human needs of compassion and understanding suppressed in place of dominant institutional practices.

*“What they see in me is like ‘oh he’s mad’*, *they’re not listening to what I was actually saying*, *so they’re not even understanding*—*they didn’t even see the post-traumatic so what they was seeing was psychosis*, *because I can hear voices”*

*(A001*: *Male*, *45–54*, *Black British)*

Denial and discreditation of experience each added to the heightened and ongoing distress experienced.

*“I got diagnosed with a bunch of different things and they were just saying oh you know I’m sure you’ll be fine*, *see us again in a couple of weeks and I was like*, *I need help now*, *like I’m- I’m just losing it*. *So that was a really frustrating time because I was just going back to the doctors again and again and not getting anywhere”*

*(C017*: *Female*, *25–34*, *White British)*

#### Discrediting voice

Participants reported being denied a voice where mental health workers were uncomfortable with speaking about what was important to the person, or where the language they used to convey their experiences and their interpretations of experiences were not aligned with those of the institutions they were speaking to.

*“Nurses don’t feel comfortable*, *mental health nurses*, *psychiatric nurses often*, *on a ward don’t feel comfortable talking about sexual relationships*, *but yet loneliness and difficulties of being in a relationship*, *finding a relationship*, *maintaining a relationship*, *the end of a relationship*… *All of these things are huge*, *have a huge impact of the wellbeing of someone and yet they’re not on the frigging table*, *explicitly*.*”*

*(B009*: *Female*, *45–54*, *Mixed—White and Black African/Caribbean*, *bisexual)*

Even when familiar with psychiatric discourse, participants felt their voices were discredited.

“*These are people I work with*! *I used to work with them*! *Coming into my house and telling me I have no insight into my mental health when I had asked those services in the first place because things had got so bad*.*”*

*(D005*: *Male*, *45–54*, *White British)*

Participants reported that they would consciously edit what they would say, how they would communicate their experiences and who they felt able to speak with for fear of judgement, stigma and reprisal.

*“It is difficult*, *you know*, *for a young woman to go through such trauma and it’s stigmatising as well*, *because sometimes it’s a bit shameful*, *you know*, *to tell people*, *oh*, *I had a mental breakdown or I’m mentally ill*, *stuff like that*. *There’s labels attached to it”*

*(A013*: *Female*, *35–44*, *African)*

#### Discrediting (multiple aspects of) identity

Participants reported on the one hand, failure to acknowledge specific aspects of identity and lived experience; and on the other, disproportionate attributions of these influences to mental health experiences.

*“It was probably quite harmful the interventions that I had there*. *There was one therapist that I saw there who was absolutely intent on pinning all of my mental health stuff to the fact that I was gay*, *rather than any of the other things that have contributed over the years”*

*(C014*: *Female*, *25–34*, *White British*, *gay)*

These intersecting phenomena however, were rarely considered together, as influencing and being influenced by the person’s overall experiences.

*“The interplay between me and accessing the service*, *and my race and identity*, *with the professional*, *that’s never taken into consideration*… *Denying that aspects of my identity has impacted my experience*, *impacted my mental health… How can you really get well when not all aspects of your identity are included*?*”*

*(B020*: *Female*, *preferred not to disclose other demographic details)*

### Outcomes of institutional injustice

#### Disengagement from formal institutions

Participants reported feelings of fear and distrust of, and disengagement from, formal institutions.

*“When talking to a clinician I would be very wary*, *I think*, *because if I say half the stuff that has happened in my head*, *you know*, *they might go*, *right*, *lock you up*, *you know”*

*(A002*: *Female*, *25–34*, *White British)*

These experiences in combination are reported to increase isolation through multiple experiences of marginalisation, and of being let down by formal institutions.

*“My history has been around drugs*. *I wanted to move away from that*, *and so it’s sort of like*, *even now I feel as if I’m left between a pillar and a post”*

*(C009*: *Male*, *45–54*, *White British)*

People reported that editing themselves and their behaviours as a means of attempting to belong had detrimental effects on the self.

*“I went through a long period of neglecting myself; my identity*, *my race*, *my culture*. *What is my culture*? *You know*. *Because of*, *you know*, *negative*, *media things and what people have said about me*. *Maybe I’m still battling with that now*. *I probably will until I go to my grave*.*”*

*(B006*: *Female*, *45–54*, *Other)*

#### Reduced affiliation with informal institutions

Participants spoke of the challenges of maintaining connections with informal institutions, when seeking help from formal institutions and working towards a greater sense of self-identity, integrity and place of belonging. Where participants experienced disparity between informal institutions and their personal sense of self, participants reported questioning their affiliations, attempting to reconcile their values whilst often adopting new ways of being in response to feeling stigmatised by families and communities.

*“I lost my dignity when I was in that relationship*, *but I am now dehumanised to living in this refuge”*

*(C019*: *preferred not to disclose demographic details)*

In attempting to seek help, participants speak of questioning their affiliations, identities and places of belonging when they are attempting to fit in to, consolidate and reconcile different ways of seeing, being and expressing themselves.

*“It does make it difficult to kind of get help or offer help because*, *you know*, *there are resources out there but if you’re kind of*, *if your community or culture doesn’t really talk about that stuff*, *then obviously it’s going to be more difficult”*

*(B021*: *Female*, *35–44*, *White and Black African/Caribbean)*

#### Negative impact on mental health and wellbeing

Participants reported cycles of marginalisation and oppression from both formal and informal institutions that negatively impacted on the individual’s mental health and well-being. As mental health and well-being deteriorated, participants reported further experiences of oppression that become increasingly difficult to escape and that made recovery more challenging.

*“That becomes problematic when you come up against oppression from people who are trying to help you*”

*(D004*: *Male*, *White British*, *preferred not to disclose age)*

As experiences worsened, participants reported being increasingly denied a voice, that their experiences were more frequently disbelieved and that these reinforced cycles of marginalisation, oppression and isolation.

*“There is something really rather sad and beautiful*, *which is that sometimes society thinks we are lying because they can’t really cope with how bad it gets”*

*(A005*: *preferred not to disclose demographic details)*

## Discussion

This study identified processes and outcomes of institutional injustice. Processes included being discredited, not being acknowledged or believed; lack of opportunity to be heard where language, voice and interpretations were different to dominant discourses; and insufficient considerations towards the complex intersecting phenomena, including lived experiences, identity and interpretations, making up the person as a whole. Harmful outcomes included: disengagement from formal institutions through fear, marginalisation and mistrust; challenges in maintaining affiliation with informal institutions when trying to consolidate new ideas and ways of being; and deteriorating mental health and well-being through experiences of continued oppression from multiple institutions.

Participants reports of having their narratives and experiences discredited is a recognised phenomenon. Fricker (2007) refers to this form of discrediting as *epistemic injustice*, distinguishing between *testimonial injustice*, relating to a lack of trust and credibility attributed to someone’s word, and *hermeneutical injustice*, arising from the lack of sensitivity, openness and responsiveness when experiences differ from one’s own [30]. The findings of this study also relates to a third type of injustice concerning identity, whereby an identifying characteristic (a ‘master status’) of a person is given disproportionate emphasis and other aspects of the person’s identity are overlooked [31]. This fragmentation ignores the intersecting characteristics and experiences of a person, rather than seeing the person as a whole. A fragmented approach influences the structure of mental health assessments as well as the results obtained, most often giving primacy to symptomatology rather than to psycho-social aspects of a person, their narratives and experiences [32]. A tendency to make judgements based on ease of available knowledge (‘heuristic bias’) can lead to errors in assessment, care and treatment through neglecting information that is available, but that might not be recalled or drawn upon as readily [33]. As demonstrated in the findings, where in giving prominence to medical discourses, a person’s culture, identity and heritage are displaced. Such bias have implications for practice, particularly the conversations conducted and the knowledge that is coproduced [32].

The findings demonstrate that formal institutions that place dominant discourses at the centre of their practices risk discrediting the roles that informal institutions play in influencing individual interpretations of lived experiences and the shaping of personal narratives [12]. A lack of meaningful consideration of the whole person can be stigmatising for an individual, as well as those they are associated with [34, 35]. The resulting self-editing and moderation of a what a person says and how a person presents, reinforces the illusion of how one is, and how one should be, in contrast to how a person really thinks and feels [34]. Superficial and inauthentic presentations of the self are harmful towards how a person behaves, and is perceived by themselves and others [22], particularly a person’s sense of identity and belonging, as revealed through the narrative findings. Minority stress, referring to the stressors embedded in the social position of minority people, takes into account the influences and effects of marginalisation upon a person’s experiences [35]. Courtesy stigma, defined as stigma by association, highlights the potential for marginalisation to occur amongst individuals, families and communities [34]. Institutional injustice limits the support and opportunities available to people and communities who are marginalised, perpetuating cycles of disadvantage. Formal institutions bias dominant discourses whilst informal institutions can foster closed communities, thus leaving marginalised people with lived experience of mental health problems vulnerable and exposed to searching for places of growth and belonging.

Institutional injustice perpetuates health inequalities and marginalised status [5, 6], as highlighted through participants’ experiences of discreditation, oppression and dehumanisation. One way to reducing inequalities is through culturally informed approaches both within existing clinical practice and by moving towards a more diverse and representative workforce at all levels [36–38]. Culturally informed approaches have the potential to reduce institutional injustice by recognising person-centred needs, addressing disparities in healthcare through considering the complex influences of biopsychosocial factors at interpersonal and institutional levels, and recognising a range of worldviews so that individual needs and preferences are not overshadowed by those of the majority [37–39]. Culturally informed approaches have the potential to improve heuristic availability through knowledge and recall of social understandings of narrative and experience [33]. In striving towards culturally informed practices however, clinicians must have the modesty not to carry out *epistemic trespass* [39] by over-reaching clinical expertise into other aspects of identity.

At the clinician level, approaches are needed to avoid two related dangers: diagnostic overshadowing in which a person’s experiences and physical symptoms are misattributed to mental illness, and granting master status to one aspect of a person’s identity by disregarding other aspects. This may require institutional transformation, with greater use of transdisciplinary approaches. For formal institutions, transdisciplinary approaches might include coproduction and peer-led services which privilege direct personal experience of phenomena. For both formal and informal institutions, a readiness to accept and be open to alternative life views are required to ensure that people who are already marginalised are not disadvantaged further. One way of achieving this is through engaging with personal narratives as a means for communicating experiences in an authentic and meaningful way, particularly in destigmatising mental health experiences [24]. An openness to ever-changing cultural diversity is needed both in clinical practice as well as approaches towards future research [40, 41]. Engagement with the narratives and perspectives of marginalised communities are needed and can only truly be understood through qualitative inquiry and where people are able to be their truly authentic selves. Further research is required into developing models of how institutional injustice occurs, particularly their contexts, influences and effects. Through these understandings, clinical change and institutional transformations can occur, towards improving the experiences of otherwise marginalised people. Institutional practices which treat, in both senses, different people differently are needed, whilst neither biasing nor excluding anyone.

## Supporting information

S1 File (DOCX)
