# Supplementary material for: Institutional injustice: Implications for system transformation emerging from the mental health recovery narratives of people experiencing marginalisation
Source: PLoS One. 2021 Apr 16;16(4):e0250367. doi: 10.1371/journal.pone.0250367 (PMC8051813; doi:10.1371/journal.pone.0250367)
Supplement: S1 File — (DOCX) [file pone.0250367.s001.docx]

**Recovery narrative topic guide**

*To be adapted by each interviewer to suit personal preference and the needs of the participant.*

**Introducing the research**

Describe the overall aims of the NEON study using the wording on the participant information sheet:

“The purpose of NEON is to understand how recovery narratives (personal stories of

mental health problems and recovery) can be of benefit to people with mental health

problems, and to design and trial a clinical intervention built around recovery

narratives”.

Explain what we mean by “recovery” here i.e. “living as well as possible, with or without symptoms”.

Describe the two aims of the interviews:

a) we want to hear stories from a range of communities that are currently under researched

b) we want to get an understanding of how stories might affect the people who hear or

read them

Explain there are two parts to the interview. We have about an hour (or 90 minutes) in total. In the

first part, we just want to hear their stories, and we’ll listen without asking any other questions (except

to clarify anything).

In the second part, we’ve got some more specific questions to ask about how stories might affect us.

Be explicitly clear that for the first part, we’re not looking for any particular type of story – there’s no

right or wrong way to talk about your experience, you can’t get it wrong, include whatever you like. It’s

also ok if it’s not in “order” or if it doesn’t feel like it’s making sense.

**Interview questions**

**PART A (UP TO 45 MINS)**

1. Can you tell me in your own words about your mental health and recovery experiences

please? For this first part of the interview, I don’t have any set questions to ask you – could

you tell me about your experience as if it were a story with a beginning, a middle, an end and

how things might look in the future? There’s no right or wrong way to tell your story - just tell

me in any way that feels most comfortable.

*Prompts: (only if narrator comes to a stand-still): Can you tell me more about that? What was*

*the experience like for you?*

*Is there anything else you’d like to tell me about your recovery story?*

**PART B (UP TO 45 MINS)**

2. (As a bridging questions from a potentially emotionally-affecting experience of sharing a

recovery story). How was that experience for you, telling me something about your story

today?

*Prompts: is it something you’re really familiar with doing, or not?*

3. How do you vary how you talk about your experience, depending on the context? E.g. who

you’re talking to, where you are, how you’re feeling that day?

*Prompts: Have you ever felt that there are parts of your story that you’re unable to share in a*

*certain context?*

4. As you know, we’re interested in how stories might affect people who hear them, and we’re

aware that sometimes it might NOT be helpful to hear a story of someone else’s experience.

Can you think of examples of times when people sharing their experiences have been

unhelpful to you?

*Prompts: Can you tell me more about this? What was unhelpful?*

*Prompts: What were your personal circumstances at the time?*

5. And can you think of examples stories that have been helpful to you?

*Prompts: Can you tell me more about this? What was helpful?*

*What was it about the person or story that had the impact? / What were your personal*

*circumstances at the time? Has this changed the way in which you give your own accounts in*

*an attempt to help others?*

*Prompts: Can you tell me more about this? What was helpful?*
